# Role of EphA4 in Mediating Motor Neuron Death in MND

**DOI:** 10.3390/ijms22179430

**Published:** 2021-08-30

**Authors:** Jing Zhao, Claire H. Stevens, Andrew W. Boyd, Lezanne Ooi, Perry F. Bartlett

**Affiliations:** 1 Queensland Brain Institute, University of Queensland, Brisbane, QLD 4072, Australia; j.zhao6@uq.edu.au; 2 Illawarra Health and Medical Research Institute, Wollongong, NSW 2522, Australia; stevensc@uow.edu.au; 3 School of Chemistry and Molecular Bioscience and Molecular Horizons, University of Wollongong, Wollongong, NSW 2522, Australia; 4 School of Medicine, University of Queensland, Brisbane, QLD 4072, Australia; andrew.boyd4@icloud.com

**Keywords:** receptors, Eph, EphA4, motor neurons, motor neuron death, neurodegenerative disease, motor neuron disease, pathogenesis

## Abstract

Motor neuron disease (MND) comprises a group of fatal neurodegenerative diseases with no effective cure. As progressive motor neuron cell death is one of pathological characteristics of MND, molecules which protect these cells are attractive therapeutic targets. Accumulating evidence indicates that EphA4 activation is involved in MND pathogenesis, and inhibition of EphA4 improves functional outcomes. However, the underlying mechanism of EphA4’s function in MND is unclear. In this review, we first present results to demonstrate that EphA4 signalling acts directly on motor neurons to cause cell death. We then review the three most likely mechanisms underlying this effect.

## 1. Introduction

Motor neuron disease (MND) refers to a group of neurodegenerative diseases, which have the shared characteristic of the progressive loss of upper and/or lower motor neurons [1,2]. Disease onset is insidious, with patients gradually losing control of their voluntary muscles, resulting in relatively late diagnosis [3]. Due to the nature of the disease and the lack of an effective treatment, MND patients usually die within 2 to 3 years following diagnosis, largely because of the loss of respiratory function [1,2,3]. Riluzole, the only drug approved in Australia, only prolongs the median life expectancy by 2 to 3 months [4,5,6,7]. New and effective therapeutic treatments are therefore urgently needed.

It is widely accepted that MND is a complex disease, with both genetic and environmental factors co-contributing to its pathogenesis [2,3,8]. Although the details of its pathogenesis are unclear, motor neuron cell death is regarded as the hallmark. Preventing this death is the primary therapeutic aim [9,10,11]. Here we discuss the importance of EphA4 signalling in motor neuron cell death, the mechanisms of signalling and the potential for ameliorating MND by blocking its signalling.

EphA4 belongs to the A subgroup of Eph receptors and is well known as a pan-receptor that can widely bind, albeit with varying affinities, to all ephrin ligands, including five glycosyl phosphatidylinositol (GPI)-linked cell membrane-bound type A ephrins and three transmembrane type-B ephrins [12,13,14]. This striking feature indicates that EphA4 interaction with both A- and B-type ephrins regulates both normal and pathophysiological functions [15]. This suggests that blocking EphA4 signalling could be more efficacious than blocking other Ephs or ephrins. Well established as a guidance molecule involved in the development of the corticospinal tract [16,17], EphA4 has also been shown to inhibit axon regeneration following spinal cord injury (SCI) [18,19,20]. The fact that EphA4 represses the axonal regrowth of motor neurons after SCI suggested that it may also contribute to the differential vulnerability of motor neurons in MND through a similar mechanism [21,22]. In support of this view, emerging research has identified that EphA4 is indeed associated with MND pathogenesis [21,22,23,24,25]. In two zebrafish models of MND, overexpressing human mutant SOD1 and TDP-43, knockdown of the zebrafish paralogue of EphA4, Rtk2, rescued both mutant SOD1-induced axonopathy and axonal outgrowth defects caused by the mutant TDP-43. The same study also examined the effect of EphA4 in rodent SOD1^G93A^ models of MND. EphA4^+/−^ mice were crossed with SOD1^G93A^ mice to obtain EphA4^+/−^ SOD1^G93A^ mice. Compared with EphA4^+/+^ SOD1^G93A^ controls, the heterozygous deletion of EphA4 significantly increased motor performance and survival. The administration of EphA4-blocking peptide to SOD1^G93A^ rats also delayed disease onset and enhanced survival. Finally, in MND patients, lower levels of expression of EphA4 mRNA in whole blood samples correlated with prolonged disease progression [21]. This study showed for the first time that EphA4 is involved in the disease progression of MND, and inhibiting EphA4 expression or activation can affect disease progression, making it an attractive target for MND therapies. Subsequently, a novel isoform of full-length EphA4 (EphA4-FL) was identified in mice and humans, EphA4-N, which contained the extracellular domains and transmembrane domain of EphA4-FL. EphA4-N was alternatively transcribed from the EphA4-FL gene, successfully translated into a functional protein, and was able to function as an endogenous dominant-negative inhibitor in terms of its repressive effect on EphA4-FL signalling in vitro. It has been shown that there was a lower level of expression of the inhibitory EphA4-N in human MND patients, compared to healthy controls, allowing more aggressive signalling by EphA4-FL. In SOD1^G93A^ mice, there was an increase in EphA4-FL expression in the pre-symptomatic phase, indicating that EphA4–FL signalling was dominant in the early pathogenesis of MND [23]. In previous studies, we generated a wildtype EphA4-Fc (a recombinant fusion protein derived from the extracellular domain of wildtype EphA4 and the Fc domain of human IgG) which effectively blocks EphA4-ephrin interaction in vitro and demonstrated that it improves functional performance in mice and rats after SCI by increasing the number of axons reaching and crossing the lesion site compared with saline-treated controls [18,19,23]. More recently, to reduce glycosylation, we mutated both the human and mouse EphA4-Fc (mEphA4-Fc) at three glycosylation sites, N235, N340 and N408. This resulted in significantly prolonging the half-life of human mEphA4-Fc from less than 24 h to 31.1 h in healthy Wistar rats following a single intravenous dose, while maintaining comparable binding and blocking ability to the ephrin ligands [26]. Results of the toxicokinetic analysis of human mEphA4-Fc cells in healthy Sprague-Dawley rats following 5× weekly repeat intravenous dosing showed that the terminal elimination half-life ranged from 52.8 h to 77.5 h. More importantly, using this glycosylation mutant of mouse mEphA4-Fc in the SOD1^G93A^ model significantly improved motor performance, including rotarod and hind-limb grip strength tests [22]. Briefly, SOD1^G93A^ mice were treated with either the mouse mEphA4-Fc or a saline control. Functional behavioural tests were monitored on a weekly basis from week 8 to the end of disease. The balance and motor coordination of mice were assessed by means of the rotarod test, whereas hind-limb grip strength was also monitored. The SOD1^G93A^ mice receiving the mEphA4-Fc treatment exhibited improved performance in the rotarod test compared to control SOD1^G93A^ mice from week 17 to week 23, with the differences at weeks 19–21 being statistically significant. Consistent with this result, mEphA4-Fc-treated SOD1^G93A^ mice also showed better hind-limb grip strength from week 8 to week 22 compared with the vehicle control group, with the differences at weeks 9 and 18–21 reaching statistical significance. Given the substantial loss of induced motor function in this model, these results suggest that mEphA4-Fc is a promising therapeutic treatment for MND and EphA4 activation is involved in the disease pathogenesis [22].

## 2. EphA4 Signalling Acts Directly on Motor Neurons to Cause Cell Death

To further investigate whether EphA4 signalling functions at the level of motor neurons to cause death in MND, SOD1^G93A^ mice with specific EphA4 gene deletion in choline acetyltransferase (ChAT)-expressing cells were obtained by crossing them with EphA4^flox/flox^ and ChAT-Cre^KI/KI^ mice on a SOD1^G93A^ genetic background [22]. ChAT is preferentially expressed in cholinergic neurons from postnatal day 5 (P5) throughout adulthood, and given that motor neurons are cholinergic neurons, the Cre-mediated deletion of the EphA4 gene was restricted to ChAT-expressing motor neurons in this mouse model [27,28]. However, as ChAT expression does not occur until P5, EphA4 expression remains normal during the critical embryonic period, allowing the normal patterning and development of the central nervous system (CNS). The results showed that heterozygous deletion of EphA4 in motor neurons significantly increased the number of surviving motor neurons in the spinal cord of the SOD1^G93A^ mice, compared to the homozygous deletion group or normal SOD1^G93A^ mice at 17 weeks of age. This was also the timepoint at which the improvement in two behavioural tasks, rotarod performance and hind-limb grip strength, reached statistical significance in the mEphA4-Fc-treated SOD1^G93A^ mice, compared with the control group. In addition, at the same timepoint, the morphology of the post-synaptic endplates of neuromuscular junctions (NMJs) in the tibialis anterior (TA) muscle were also better maintained in the heterozygous deletion group at the same timepoint, and more fragmented post-synaptic endplates of NMJs and debris of damaged endplates were observed in another two control groups [22]. This is the first evidence to reveal that EphA4 exerts direct negative effects on motor neuron survival in MND. Notably, only SOD1^G93A^ mice with specific heterozygous deletion of EphA4 in motor neurons exhibited a significant protective effect on cell survival, with the motor neuron death in the homozygous deletion group being similar to that in normal SOD1^G93A^ controls [22]. However, SOD1^G93A^ mice with homozygous germline deletion of EphA4 showed reduced viability due to a low birth rate and body weight [21]. Taken together, these data suggest that although a certain expression level of EphA4 is essential for motor neuron development and survival, a lower level of expression of EphA4 signalling could protect motor neurons from death in MND.

To further investigate the direct effect of EphA4 on motor neurons, we have first estimated the expression level of EphA4 in motor neurons using a human-induced pluripotent stem cell (iPSC)-derived motor neuron system [18,29]. Fibroblasts were obtained via skin biopsy from a symptomatic familial MND patient harbouring a SOD1^E101G^ mutation and a healthy control [30]. The methods were carried out in accordance with the guidelines set out in the National Statement on Ethical Conduct in Research Involving Humans and informed consent was obtained from all donors [31]. All experimental protocols were approved by the University of Wollongong Human Research Ethics Committee (approval HREC 13/272, most recently reviewed and approved on 29 June 2021). Fibroblasts were reprogrammed into iPSCs and confirmed as pluripotent, as previously described [29]. The iPSCs were cultured on Matrigel-coated 6-cm tissue culture plates in TeSR-E8 (Stem Cell Technologies) at 37 °C, 5% CO_2_, 3% O_2_ in a humidified incubator (hypoxic conditions). The iPSCs were differentiated into motor neurons as previously described [29], with some modifications. Neuronal precursor cells were treated with increasing concentrations of retinoic acid over three days (0.1 µM to 0.3 µM), and on the third day 2 µM of purmorphamine was also added to the medium [32]. BrainPhys (Stemcell Technologies) was used as the basal medium to generate motor neuron precursor cells and mature motor neurons [33].

**Figure 1:**
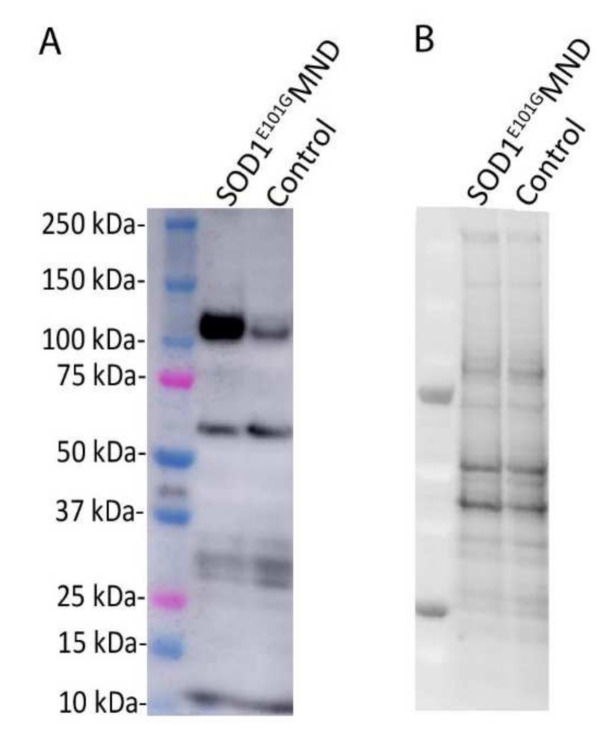
(**A**) Relative levels of EphA4 (110 kDa) are 5× higher in iPSC-derived motor neurons from a SOD1^E101G^ MND patient compared to iPSC-derived motor neurons from a healthy control. (**B**) Corresponding total protein blot from the SOD1^E101G^ MND patient and healthy control. Four-week-old iPSC-derived motor neurons were harvested by rinsing in 1× PBS followed by manual scraping in RIPA buffer (50mM Tris-HCl pH 7.4, 1% NP40, 0.25% Na-deoxycholate, 150 Mm NaCl, 1mM EDTA, phosphate (PhosSTOP, Roche) and protease (complete protease inhibitor cocktail, Roche) inhibitors). Cellular debris was pelleted by means of centrifugation at 10,000× *g* for 10 min at 4 °C. The cleared protein lysate was quantified via BCA and 20 µg of protein was used for Western blotting. Proteins were denatured by boiling at 95 °C for 5 min in Laemmli buffer with 5% β-mercaptoethanol and loaded on a Criterion 4–20% stain-free gel (Biorad). Samples were electrophoresed in SDS-PAGE buffer and transferred onto a PVDF membrane. The membrane was imaged using the Criterion stain-free gel imaging system (Biorad) to obtain total protein values for quantification. The membrane was blocked in 5% skim milk in TBS for 1 h and incubated in EphA4 antibody (1:1000 in 5% skim milk in Tris-buffered saline solution, mouse anti-EphA4, ECM Bioscience) overnight at 4 °C. The membrane was then incubated in an HRP-conjugated secondary antibody and EphA4 (approximate molecular weight 110 kDa) was detected using Pierce ECL Plus Western Blotting substrate (Thermo Fischer) and the chemiluminescence function on the Amersham GE 600 Imager (GE Life Sciences). Densitometry analysis was conducted using Image Studio Lite version 5.2 and each sample measurement was normalised to its corresponding total protein value.

After one week of proliferation and three weeks of maturation under hypoxic conditions, the relative levels of EphA4 protein were compared between MND and healthy control iPSC-derived motor neurons using Western blotting. The BCA and Western blot assays were conducted as described previously [23], except that 20 µg of protein was used for the Western blotting. Each sample measurement was normalised to its corresponding total protein value and the relative level of EphA4 protein was five times higher in iPSC-derived motor neurons from the SOD1^E101G^ MND patient compared to motor neurons derived from the healthy control (Figure 1).

**Figure 2:**
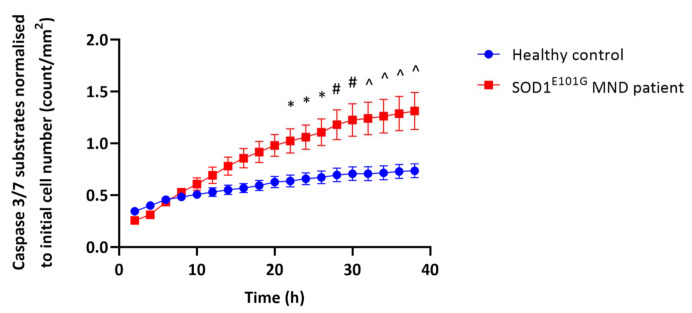
IPSC-derived motor neurons from a SOD1^E101G^ MND patient are more vulnerable to oxidative stress then iPSC-derived motor neurons from a healthy control. IPSC-derived motor neurons from a SOD1^E101G^ MND patient and a healthy control were matured for three weeks under hypoxic conditions (3% oxygen) and then transferred to a normoxic incubator (21% oxygen) for 38 h in order to induce oxidative stress. Apoptosis was assessed by quantifying the number of caspase 3/7 substrates every 2 h using an Incucyte. Two-way ANOVA revealed that there were significant main effects of time (F(18,144) = 36.53, *p* < 0.0001), cell line (F(1.8) = 8.677, *p* = 0.0185), and time × cell line interaction (F(18.112) = 8.830, *p* < 0.0001). Sidak’s test for multiple comparisons showed that iPSC-derived motor neurons from the SOD1^E101G^ MND patient showed a significant increase in the number of caspase 3/7 substrates per mm^2^ from 22 h onwards compared to the healthy control. Data presented as mean ± SEM of *n* = 5 technical replicates; * *p* < 0.05; # *p* < 0.01; ^ *p* < 0.001.

To gain insight into the role of highly expressed EphA4 in iPSC-derived motor neuron death in MND, we next determined whether the iPSC-derived motor neurons from the MND patient carrying a SOD1^E101G^ mutation were more vulnerable to oxidative stress, the most common stress observed under MND conditions, than iPSC-derived motor neurons from a healthy control [34,35]. As previously described, iPSC-derived neurons transitioned from hypoxic to normoxic culture conditions can be used to assess the impact of oxidative stress on caspase 3/7 activity, as a marker of apoptosis [30]. After a maturation phase under hypoxic conditions, iPSC-derived motor neurons were transferred to a humidified incubator at 37 °C, 5% CO_2_ and 21% O_2_ (normoxic conditions). At the time of transfer, NucView 488 caspase 3 enzyme substrate (which fluoresces in response to caspase 3/7 activity in cells, Gene Target Solutions) and Reddot 1 (live cell marker, Gene Target Solutions) were added to the motor neuron culture. Caspase 3/7 activity was assessed every two hours for 38 h using an Incucyte (Essen Bioscience) instrument. From 22 h, the levels of caspase 3/7 activity were significantly higher in the iPSC-derived motor neurons from the MND patient compared to the iPSC-derived motor neurons from the healthy control (Figure 2). This suggests that the iPSC-derived motor neurons from the MND patient harbouring a SOD1^E101G^ mutation were more vulnerable to apoptotic cell death under these oxidative stress conditions compared to control. Together, these findings led us to ask if the increased EphA4 expression could contribute to the vulnerability of MND motor neurons under stress conditions.

**Figure 3:**
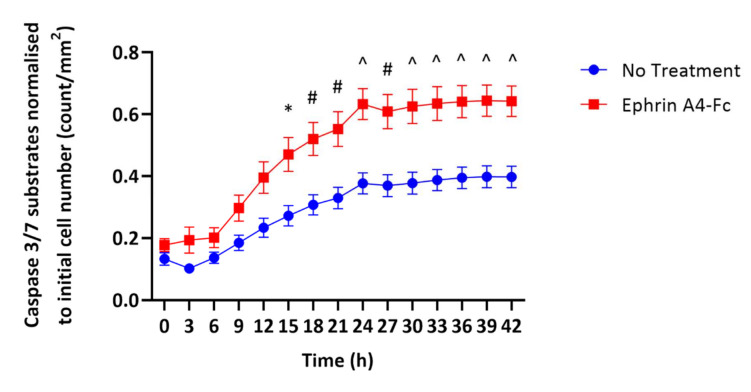
Treatment with the EphA4 ligand, ephrin A4-Fc results in increased cell death in iPSC-derived motor neurons from a SOD1^E101G^ MND patient. IPSC-derived motor neurons from a SOD1^E101G^ MND patient were matured for four weeks under hypoxic conditions (3% oxygen) and then transferred to a normoxic incubator (21% oxygen) for 42 h, with or without the EphA4 ligand ephrin A4-Fc (10 µg/mL). Apoptosis was assessed by quantifying the number of caspase 3/7 substrates every 3 h using an Incucyte. Two-way ANOVA revealed that there were significant main effects of time (F (14.112) = 219.2, *p* < 0.0001), treatment (F (1.8) = 11.86, *p* = 0.0088), and time × treatment interaction (F (14.112) = 14.46, *p* < 0.0001). Sidak’s test for multiple comparisons showed that iPSC-derived motor neurons from the SOD1E101G MND patient treated with ephrin A4-Fc showed a significant increase in the number of caspase 3/7 substrates per mm^2^ from 15 h onwards compared to motor neurons without treatment. Data presented as mean ± SEM of *n* = 5 technical replicates; * *p* < 0.05; # *p* < 0.01; ^ *p* < 0.001.

To address this possibility, we next investigated whether the activation of the EphA4 induced by ephrin ligands led to apoptosis in MND motor neurons. Four-week-old iPSC-derived motor neurons from the SOD1^E101G^ MND patient were transferred from hypoxic (3% O_2_) to normoxic (21% O_2_) conditions. At the time of transfer, NucView 488 caspase 3 substrate and Reddot 1 were added to the cells as described above, either with or without ephrin A4-Fc ligand (10 µg/mL). Caspase 3/7 activity was assessed every 3 h for 42 h using the Incucyte. From 15 h, the levels of caspase 3/7 activation were significantly higher in the iPSC-derived motor neurons from the SOD1^E101G^ MND patient treated with ephrin A4-Fc compared to motor neurons without treatment (Figure 3). This suggests that EphA4 activation may directly contribute to motor neuron death in MND. In addition, lactate dehydrogenase (LDH) levels in the cell culture medium were measured using a Pierce LDH Cytotoxicity Assay Kit (Thermo Scientific, catalog #88953). LDH cytotoxicity (A490 nm–A680 nm and normalised to the total protein values from each line) was two times higher in the ephrin A4-Fc-treated motor neurons compared to those with no treatment (0.08 vs. 0.04). Altogether, these data suggest that the activation of overexpressed EphA4 in iPSC-derived motor neurons from an MND patient directly results in increased motor neuron death.

## 3. Direct Regulation of Motor Neuron Death by EphA4

So far, we have observed the directly negative effect of EphA4 activation on motor neuron survival upon both in vitro and in vivo MND backgrounds (Figure 1, Figure 2 and Figure 3 and [22]). These are confirmatory evidence to support the promotive effect of EphA4 activation and the protective effect of EphA4 deletion or inhibition on MND progression. Although EphA4 activation has been directly or indirectly involved in different types of cell death, such as NIH 3T3 cells, glioblastoma multiform tumoral cells, retinal ganglion cells and endothelial cells [36,37,38,39], this was the first time that the novel effect of EphA4 on motor neuron survival in MND had been reported. More investigations are required to explicate the underlying mechanisms. In the following review, three possible mechanisms are concisely discussed.

### 3.1. Role of Rho/Rock Signalling

To date, more than 10 different types of cell death have been identified, and four major types among them are apoptosis, necrosis, autophagy and entosis [40]. Although motor neuron death in MND is likely to be multifactorial, the final demise of these cells is more likely to occur via a programmed, energy-dependent cell death pathway resembling apoptosis [41]. It is widely accepted that typical morphological changes which occur during the cell apoptosis process include the release of apoptotic bodies, plasma membrane blebbing and nuclear condensation [42]. Molecules regulating these processes thus played important roles in regulating cell apoptosis [43,44,45,46,47,48]. One of the critical regulators of apoptotic cell membrane blebbing is Rho-associated coiled-coil-containing protein kinase (ROCK), which is also part of the EphA4 downstream signalling pathway [25,38,49,50]. ROCK activation has been shown to contribute to membrane blebbing during the eosinophil peroxidase-induced death of lung epithelial cells in vitro [43]. Similarly, in a human umbilical vein endothelial cell (EC) culture system, the application of combretastatin A-4-phosphate (CA-4-P), a tumor vascular-targeting agent, significantly enhanced ROCK signalling activation and its subsequent myosin light chain (MLC) phosphorylation, which were shown to be responsible for the cell membrane blebbing, loss of cell adherence and decreased viability of ECs due to reorganisation of the actomyosin cytoskeleton. These results suggest that this mechanism may underlie the effect of the CA-4-P treatment in promoting tumor EC death and leading to the shutdown of blood flow in tumors in vivo [44]. ROCK and its other substrates have also been reported to regulate the cell death process [45,46,47]. The upregulation of ROCK signalling and the subsequent inhibition of downstream mitogen-activated protein kinases (MAPK) signalling has been shown to promote the loss of cell bipolarity and detachment, resulting in increases in EC death in an EC-fibroblast coculture system in vitro and in xenograft tumors in nude mice growing from a human colorectal cancer cell line in vivo [45]. Another identified ROCK substrate, phosphatase and tensin homologue (PTEN), has been shown to be involved in the protective effect of ROCK inhibition on cardiomyocyte apoptosis [46] and EC survival in the cardiovascular system in vitro [47]. Activation of ROCK signalling is also highly likely to be involved in regulating motor neuron apoptosis in MND. In support of this, Takata and colleagues revealed that inhibition of ROCK by Fasudil or Y-27632 prevented motor neuron death in mouse motor neuron (NSC34) cell cultures and in the lumbar anterior horn in the SOD1^G93A^ mouse model [48]. Moreover, these two ROCK inhibitors were shown to delay disease onset, prolong the mean survival time and improve functional performance in the SOD1^G93A^ mouse model [48,51]. A multicentric, double-blind, randomised, placebo-controlled phase 2a clinical trial of Fasudil in 120 MND patients started in early 2019, aiming to assess its safety, tolerability and efficacy (ROCK-ALS trial, NCT03792490, Eudra-CT-Nr.: 2017-003676-31) [52]. In addition, three MND cases have been reported in which fasudil was compassionately used for treatments from 2017 to 2019, demonstrating good tolerance [53]. However, it is unclear how ROCK signalling is activated/regulated in the pathogenesis of MND.

Considering that ROCK is part of the EphA4 downstream signalling pathway, the correlation of high expression of the EphA4 receptor with rapid disease progression of MND is consistent with the idea that EphA4 mediates this effect through increased ROCK signalling. The EphA4 receptor tyrosine kinase is expressed on the cell surface and exerts diverse effects through the activation of multiple downstream signalling pathways [54,55]. Following the activation of EphA4, the GTPase Rho family, including Rho, Rac1 and Cdc42, is activated, interacting with its downstream effector proteins to exert different effects [20,50,56,57,58]. ROCK is one of the major effectors for Rho, and their interaction induces conformational changes of Rho and activates ROCK [59]. It has been reported that EphA4 negatively affects axon regeneration after SCI primarily through the Rho/ROCK signalling pathway [20], and the administration of Y-27632 has been shown to increase dendritic branching and axonal regeneration [60]. Similarly, the expression of EphA4 is significantly increased following ischemia-reperfusion in vitro and in vivo, and the activation of EphA4 contributes to the disruption of the blood–brain barrier (BBB) post-ischemic brain injury through Rho/ROCK signalling [38]. In the subarachnoid hemorrhage (SAH) rat model, EphA4 activation has also been shown to be responsible for the neuronal apoptosis and BBB breakdown through the ROCK pathway [49]. Moreover, a reduction in EphA4 has been shown to improve the behavioural function of different ischemic stroke animal models via the inhibition of its downstream Rho/ROCK pathway [38,49,50]. Therefore, it is likely that during MND progression, high expression of EphA4 on motor neurons participates in the increasing motor neuron death by activating the Rho/ROCK downstream pathway.

### 3.2. Role of D-Serine-Induced NMDAR Activation

Excitotoxicity has been reported to contribute to neuronal death in MND [61]. One classical form of excitotoxicity is induced by excessive stimulation of the *N*-methyl-d-aspartate receptor (NMDAR), which is potentially caused by elevated levels of its glutamate ligand and/or d-serine co-agonist [62,63] and eventually resulting in apoptosis or necrosis in neurons [64,65]. Endogenous d-serine largely contributes to NMDAR-mediated neurotoxicity, with a decrease in d-serine levels being shown to repress this excitotoxicity [66]. Recently, d-serine has been reported to be involved in MND progression [67,68,69], providing the first direct evidence that increased concentrations of d-serine occur in the brain and spinal cord of MND patients and SOD1^G93A^ mice, and that, as a key determinant of glutamate toxicity, this leads to motor neuron degeneration [67,68]. Given that Zhuang et al. have revealed that normal EphA4 function facilitates the synthesis and/or release of d-serine in the adult brain in vitro [70], the EphA4 dysfunction-induced changes in the d-serine concentration are also likely to contribute to motor neuron death via NMDAR dysfunction. However, further investigation is required to address how EphA4 regulates the d-serine levels in the brain in vivo, and which source of d-serine (astrocytes and/or neurons) has been affected. This EphA4-regulated d-serine concentration has already been observed during adult hippocampal neurogenesis in vivo and in vitro [71]. We have shown that either pharmacological EphA4 inhibition by EphA4-Fc or genetic deletion of EphA4 upregulates adult hippocampal neural precursor proliferation. Moreover, this increased precursor activity induced by the inhibition of EphA4 could be rescued by exogenous d-serine supplementation. In addition, a specific blockage of the interaction between d-serine and NMDARs promoted precursor proliferation directly [71]. These results suggest that EphA4 inhibition indirectly leads to an increase in adult precursor proliferation, by decreasing the level of d-serine, thus inducing downregulation of NMDARs, which directly exerted positive effects on precursor proliferation. Likewise, the high expression of EphA4 in motor neurons might contribute to the increased concentration of d-serine in MND, which results in increased motor neuron death through upregulating NMDAR-induced excitotoxicity.

d-serine is endogenously converted from l-serine by serine racemase (SR), and degenerates to keto acid via the activity of d-amino acid oxidase (DAO). Both increased activity of SR and the loss of DAO activity have also been observed in SOD1^G93A^ mice [67,68]. A loss-of-function mutation in the DAO (R199W DAO) gene has also been associated with familial MND cases. Subsequent research revealed that the expression of R199W DAO promotes the accumulation of d-serine, which led to cell death in primary motor neuron cultures [69,72]. So far, these changes in SR and DAO have been observed in familial MND cases and transgenic mouse models, but they may also exist in sporadic MND cases and co-regulate the concentration of d-serine with EphA4 activation. This may be one of the reasons why EphA4 inhibition only partially improves functional behavioural performance in MND mouse models.

### 3.3. Role of Calcium Channels

Many studies have reported that changes in ion channels and ion homeostasis lead to increases in neuronal firing, oxidative stress and mitochondrial dysfunction, all of which could eventually contribute to apoptotic cell death [73] and be involved in MND pathology [74,75,76,77]. These abnormalities have mainly been attributed to increased Na^+^, decreased K^+^ and increased Ca^2+^ fluxes [78]. Among other effects, EphA4 activation is likely to regulate the intracellular Ca^2+^ concentration. As mentioned above, high EphA4 expression is likely to upregulate the function of NMDARs expressed in motor neurons by increasing the levels of d-serine. Subsequently, the activation of NMDARs results in opening of the ion channel, which causes excessive excitotoxic influx of Ca^2+^ to motor neurons in MND [79]. AMPA receptors (AMPARs) are also well-known ionotropic glutamate receptors, the activation of which mediates a massive influx of Ca^2+^, leading to excitotoxicity in MND pathology [61,80]. In the SOD1^G93A^ mouse model, the expression of the GluA2 subunit of AMPARs is significantly lower than that in normal mice (which mainly upregulates Ca^2+^ permeability) [81,82]. Higher expression of the GluA3 subunit has been found to accompany this decreased expression of the GluA2 subunit in SOD1^G93A^ mice, with implications for disease onset and progression [82]. Another study reported the upregulation of the GluA1 subunit, rather than the GluA3 subunit, and downregulation of the GluA2 subunit in the highly expressed Ca^2+^-permeable AMPARs in iPSC-derived motor neurons carrying human *C9orf72* and patient primary motor neurons [83]. This increase in Ca^2+^-permeable AMPAR activity caused a decline in motor neuron function and accelerating cell death, which is consistent with MND progression [84]. Given that EphA4 activation leads to the degradation of surface AMPARs at synapses [85,86] and dendritic spine loss [87,88], it is possible that EphA4-regulated changes in AMPARs contribute to altered Ca^2+^ currents that cause progressive motor neuron death in MND.

## 4. Conclusions

Research to date suggests that EphA4 dysfunction in motor neurons likely contributes to progressive cell death directly in MND pathology. However, the underlying mechanism remains unclear. Here, we raise three potential mechanisms: EphA4 downstream Rho/ROCK signalling activation, EphA4-mediated d-serine-related NMDAR dysfunction and EphA4-dependent altered Ca^2+^ currents. Further investigations are required to determine which mechanism plays the major role in regulating the EphA4-induced effect on motor neuron death in MND. Considering that EphA4 can bind to almost all ephrin ligands, different types of ligand-induced EphA4 activation may exert the same effect on motor neuron survival with different preferences for mechanisms, depending on the tissue- and cell-type specificity. These findings also indicate that mEphA4-Fc is likely to be an effective therapy for MND, due to its ability to competitively bind all EphA4 ligands. The related research into this mechanism could provide insight into combination drug therapies to further improve the therapeutic outcomes for MND patients.

## Data Availability

Data is contained within the article.

## Abbreviation

MND	Motor neuron disease
GPI	Glycosyl phosphatidylinositol
SCI	Spinal cord injury
EphA4-FL	Full-length EphA4
mEphA4-Fc	Mutant EphA4-Fc
ChAT	Choline acetyltransferase
P5	Postnatal day 5
CNS	Central nervous system
NMJ	Neuromuscular junction
TA	Tibialis anterior
iPSC	Induced pluripotent stem cell
LDH	Lactate dehydrogenase
ROCK	Rho-associated coiled-coil-containing protein kinase
EC	Endothelial cell
CA-4-P	Combretastatin A-4-phosphate
MLC	Myosin light chain
MAPK	Mitogen-activated protein kinases
PTEN	Phosphatase and tensin homologue
BBB	Blood–brain barrier
SAH	Subarachnoid haemorrhage
NMDAR	*N*-methyl-d-aspartate receptor
SR	Serine racemase
DAO	D-amino acid oxidase
AMPAR	AMPA receptor
